# NLRP3 Inflammasome Is Involved in Calcium-Sensing Receptor-Induced Aortic Remodeling in SHRs

**DOI:** 10.1155/2019/6847087

**Published:** 2019-02-13

**Authors:** Xin Zhang, Siting Hong, Shuhan Qi, Wenxiu Liu, Xiaohui Zhang, Zhiyu Shi, Wenjia Chen, Meng Zhao, Xinhua Yin

**Affiliations:** Department of Cardiology, First Affiliated Hospital of Harbin Medical University, Harbin, 150001 Heilongjiang, China

## Abstract

Increasing evidence suggests that the NLRP3 (nucleotide oligomerization domain-like receptor family, pyrin domain containing 3) inflammasome participates in cardiovascular diseases. However, its role and activation mechanism during hypertension remains unclear. In this study, we tested the role and mechanism of calcium-sensing receptor (CaSR) in NLRP3 inflammasome activation during hypertension. We observed that the expressions of CaSR and NLRP3 were increased in spontaneous hypertensive rats (SHRs) along with aortic fibrosis. In vascular smooth muscle cells (VSMCs), the activation of NLRP3 inflammasome associated with CaSR and collagen synthesis was induced by angiotensin II (Ang II). Furthermore, inhibition of CaSR and NLRP3 inflammasome attenuated proinflammatory cytokine release, suggesting that CaSR-mediated activation of the NLRP3 inflammasome may be a therapeutic target in aortic dysfunction and vascular inflammatory lesions.

## 1. Introduction

Hypertension, a threat to human health, is a complex disease that can cause end organ damage associated with vascular remodeling, which is characterized by growth, apoptosis, inflammation, and fibrosis [1]. Vascular remodeling, depending on the function of vascular smooth muscle cells (VSMCs) and homeostasis of extracellular matrix in the arterial wall, closely correlates with the activation of the renin angiotensin aldosterone system (RAAS), the activity of matrix metalloproteinase (MMP), and the release of inflammatory mediators and cytokines [2]. However, the molecular mechanisms responsible for vascular remodeling in hypertension remain to be determined.

Increasing evidence indicates that the inflammation and immune system activation, including proinflammatory cytokines such as interleukin (IL) and immune cells like lymphocytes, play a critical role in cardiovascular diseases, vascular injury, and VSMC phenotypic modulation and dysfunction [3, 4]. The NLRP3 inflammasome, a key signaling platform that activates highly proinflammatory cytokines, IL-1*β* and IL-18, contributes to the development of aortic aneurysms and hypertension via vascular inflammation [5, 6]. Activation of NLRP3 promotes the formation of the NLRP3 inflammasome complex, comprising NLRP3, apoptosis associated speck-like protein containing a caspase recruitment domain (ASC) and caspase 1 [7], which leads to cell injury and dysfunction in a caspase 1-dependent manner [6, 8]. However, the activation mechanisms of the NLRP3 inflammasome complex and its roles in aortic remodeling in hypertension are largely unknown.

CaSR, a seven-transmembrane helical domain (7TMD) and G protein-coupled receptor that senses the extracellular calcium concentration, is functionally expressed in the parathyroid, kidneys, bone, skin, stomach, and vessels [9, 10]. Previous studies have reported that CaSR participates and plays an important role in cell proliferation, apoptosis, and inflammation [11–13]. CaSR and its allosteric modulator play an important role in VSMC function [14, 15]. It has been reported that CaSR can activate the NLRP3 inflammasome, amplifying the inflammation response, which is mediated by increased intracellular inositol phosphate/Ca^2+^ pathway in monocytes and macrophages [13, 16], but its role in aortic remodeling remains to be elucidated. The purpose of this study was to investigate the role and potential mechanisms of CaSR in aortic remodeling during hypertension.

## 2. Materials and Methods

### 2.1. Materials and Reagents

Calhex 231 hydrochloride (Calhex 231, SML0668), angiotensin II (Ang II, A9525), cytokine release inhibitory drug 3 (CRID3, CP-456773), and BAPTA/AM (A1076) were purchased from Sigma-Aldrich (St. Louis, MO, USA). Calindol hydrochloride (calindol, sc-211006) and an antibody against ASC (sc-22514R) were purchased from Santa Cruz Biotechnology (Santa Cruz, CA, USA). An antibody against CaSR (ACR-004) was acquired from Alomone Labs Ltd. (Hadassah Ein Kerem, Jerusalem). Antibodies against NLRP3 (bs-10021R) and IL-1*β* (bs-0812R) were purchased from Bioss (Beijing, China). An antibody against IL-18 (PAB16177) was purchased from Abnova (Taipei, Taiwan). An antibody against pro-IL-1*β* was obtained from Proteintech (Wuhan, Hubei, China). Antibodies against TIMP2 (ab64040), MMP2 (ab92536), MMP9 (ab76003), collagen I (ab34710), collagen III (ab7778), and caspase 1 (ab179515) were purchased from Abcam Inc. (Cambridge, MA, USA). Fluo-3/AM (S1056) was purchased from Beyotime Biotechnology (Shanghai, China). An antibody against GAPDH (TA-08) and all secondary antibodies were obtained from ZSGB-Bio (Beijing, China). All other chemicals and reagents were of analytical grade.

### 2.2. Animals and Tail Cuff Measurements

Specific pathogen-free, male inbred SHRs and WKY rats were purchased from Vial River Laboratories (Beijing, China). Animals were studied at 20 weeks of age and divided into 3 groups: WKY rats group, SHRs treated with injections of saline (vehicle, ip, 28 d, *n* = 15), and SHRs treated with Calhex 231 (10 *μ*mol/kg·d, ip, 28 d, *n* = 10). The blood pressure of age-matched animals was measured by tail cuff while animals were awake and during daytime using a BP2010A blood pressure system (Softron Biotechnology Co. Tokyo, Japan). An average of the arterial blood pressure for at least 10 cycles was taken for each animal and then averaged within the group. All animal care and experimental protocols were in accordance with the Institutional Animal Care and Use Committee of Harbin Medical University, China.

### 2.3. Cell Culture

Rat VSMCs were acquired from the thoracic aorta of male Wistar rats with an explant method as previously described [4, 17]. Then, they were seeded in DMEM, 10% FBS at 37°C and 5% CO_2_. VSMCs at passages 3~6 and 80-90% confluence were used in our experiments. Cell growth was arrested by incubating the cells in serum-free DMEM for 24 h prior to use. The VSMCs were treated according to the experimental plan.

### 2.4. Western Blot

After treatment, all experimental samples in vitro and in vivo were collected and were homogenized in lysis buffer on ice, which contained 1% proteinase inhibitor solution. Then, the homogenates were centrifuged at 13,500 rpm for 20 min at 4°C and the supernatant obtained by centrifugation was proteins extracted from samples. The total protein concentrations were measured with a BCA protein assay kit (Beyotime Biotechnology, Shanghai, China). Same amounts of total protein were separated by electrophoresis on 10% or 12.5% SDS-PAGE gels before they were transferred onto PVDF membrane as previously described [16]. After blocked with 5% nonfat dry milk (1 h, room temperature), membranes were incubated overnight at 4°C in primary antibodies diluted in a 5% bovine serum albumin solution in TBST. The antibodies used for western blot analysis are as follows: CaSR (1 : 500), NLRP3 (1 : 300), ASC (1 : 200), caspase 1 (1 : 1000), IL-18 (1 : 300), IL-1*β* (1 : 500), pro-IL-1*β* (1 : 500), MMP2 (1 : 1000), MMP9 (1 : 1000), TIMP2 (1 : 1000), collagen I (1 : 5000), collagen III (1 : 5000), and GAPDH (1 : 1000). The samples were then treated with secondary antibody for 1 h at room temperature and developed with the ECL detection system. The films were scanned and analyzed using Bio-Rad Image Lab software.

### 2.5. Immunohistochemistry Staining

To determine the expression of CaSR and infiltration of macrophage in the aortas, immunohistochemistry staining was performed. Briefly, the aorta tissues were fixed in 4% formalin and embedded in paraffin. Then, samples were cut into sections (3 *μ*m) for use. The sections were blocked in goat serum blocking solution at room temperature for 20 min after deparaffinized, rehydrated, blocked with 3% H_2_O_2_, and washed with 0.1 M PBS. The sections were incubated with primary antibody at 4°C overnight. The sections were incubated with primary antibody at 4°C overnight. Antibodies used in this study were as follows: CaSR (1 : 100), CD68 (1 : 50, Abcam), CD11b (1 : 400, Abcam), and CD206 (1 : 100, Santa Cruz). After washing with PBS, the sections were incubated with secondary antibody at room temperature for 30 min. 3,3-Diaminobenzidine was used for color development in arteries. Sections were then counterstained with hematoxylin, incubated in ammonia, dehydrated with gradient ethanol, transparentized with xylenes, and covered with glass coverslips with neutral resin. Cells with brown-strained particles were denoted as positive based on imaging with a microscope (Zeiss, Germany). Quantitative assessments were performed with Image-Pro Plus software. An average of the number of positive cells for at least 5 random areas was taken for each section and then averaged within the group.

### 2.6. Masson Trichrome Staining

Following anesthesia, vessel segments (5 mm) were excised and placed in 4% paraformaldehyde fixing solution (24 h, 4°C) and then embedded in paraffin with routine methodology. The dissected arteries (5 *μ*m) were stained with Masson trichrome stain for fibrosis assessment. Images (5 per ring) for the aortic sections were captured using a Zeiss microscope. Blue-dyed collagen fibers were considered to be fibrosis. The fibrosis area was measured with Image-Pro Plus Software. The level of fibrosis was represented by the relative ratio of fibrosis area to vascular area.

### 2.7. IL-1*β* and IL-18 Concentration Measurement

Levels of IL-1*β* and IL-18 in cell supernatant were measured using ELISA kits (BlueGene Biotech, Shanghai, China) according to the manufacturer's instructions. Optical density was read at 450 nm using a microplate reader (BioTek, USA).

### 2.8. Measurement of Cytosolic [Ca^2+^]_i_ in VSMCs

VSMCs were loaded with fluo-3/AM (5 *μ*M) for 15 min (37°C) and washed with Hanks' balanced salt solution for 4 times. The fluorescence images were captured by using a fluorescence microscope (Zeiss, Germany). The levels of cytosolic [Ca^2+^]_i_ in VSMCs were represented by increased intensity of fluorescence.

### 2.9. Statistical Analysis

All experiments were repeated more than three times. Data are expressed as the mean ± SD. And if they are normally distributed, the differences between two groups were evaluated using the *t*-test; significance between multiple groups was evaluated by one-way ANOVA and followed by Tukey's test. If they are not, Mann-Whitney test was used for 2 groups, and Kruskal-Wallis test was used for more than 3 groups. A *P* value < 0.05 was considered statistically significant.

## 3. Results

### 3.1. Effect of CaSR on the Blood Pressure Levels in SHRs

Considerable evidence has shown that CaSR regulates blood vessel tone and blood pressure [14, 18]. However, the mechanism is not entirely clear. To directly test the effect, we examined the effect of CaSR on the blood pressure levels in SHRs, including the systolic blood pressure (SBP), diastolic blood pressure (DBP), and mean arterial blood pressure (MAP). As shown in Table 1, compared with WKY rats, the blood pressure levels of SHRs were increased. A comparable level of reduction was observed in SHRs when treated with Calhex 231, an inhibitor of CaSR, for 4 weeks, but the blood pressure levels remain significantly higher than in WKY rats. These results indicated that CaSR could regulate the levels of blood pressure in SHRs. However, there was no statistical difference in heart rate and body weight among these three groups.

### 3.2. Inhibition of CaSR Attenuated NLRP3 Inflammasome in the Aortas from SHRs

**Figure 1:**
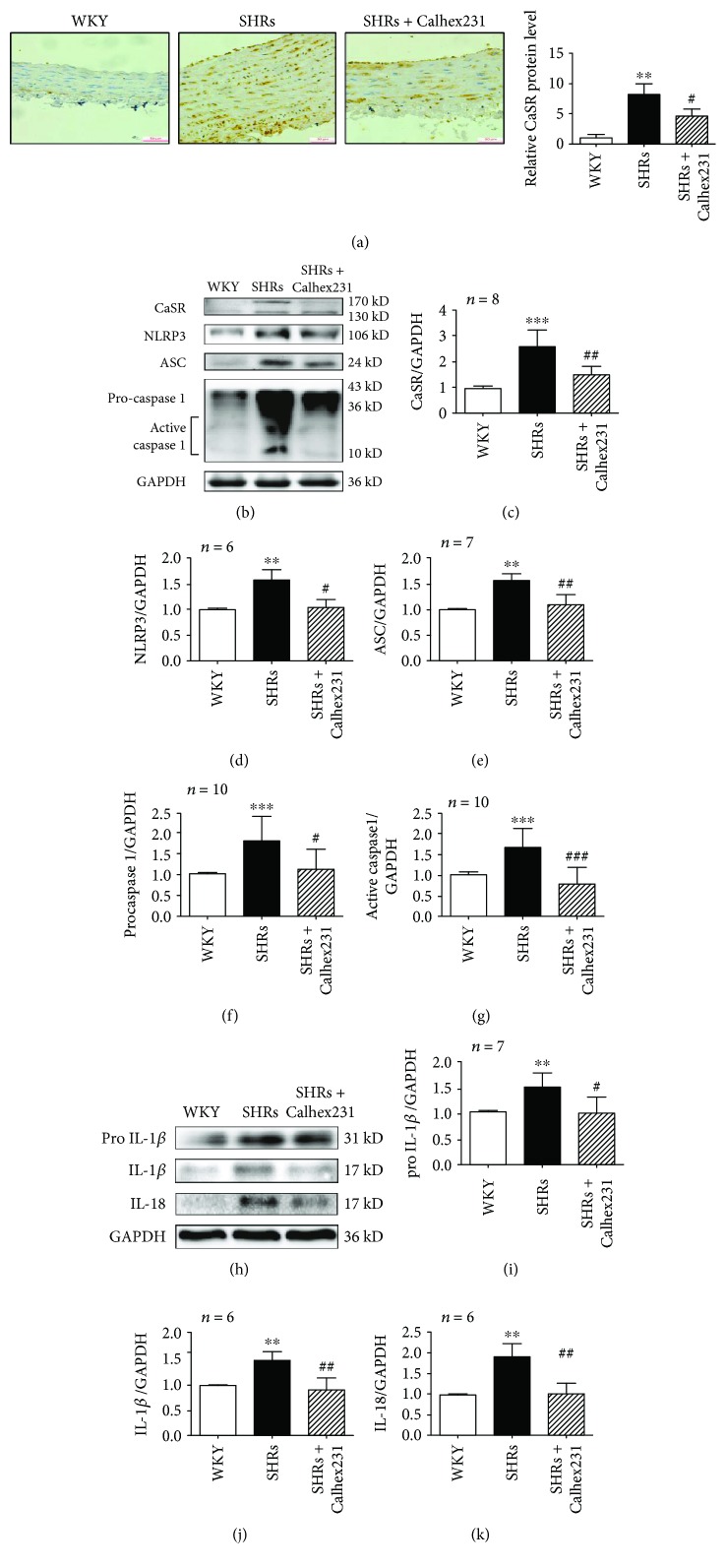
Calhex 231 attenuated NLRP3 inflammasome activation in the aortas of SHRs. (a) Immunohistochemistry was used to detect CaSR expression in the aortas of rats. Quantification of the relative CaSR protein levels in these arteries demonstrated that CaSR was increased in SHRs and decreased by antagonist of CaSR, bar = 50 *μ*m, *n* = 5. (b) Western blot analysis of CaSR, NLRP3, ASC, and caspase 1. (c–g) Bar graphs showed the quantitative analyses of the proteins from B. (h) Representative western blot images of pro-IL-1*β*, IL-1*β*, and IL-18 in the aortas from experimental rats. (i–k) Quantitative analyses of proteins from in H. ^∗∗^ *P* < 0.01 and ^∗∗∗^ *P* < 0.001 vs. WKY; ^#^ *P* < 0.05, ^##^ *P* < 0.01, and ^##^ *P* < 0.001 vs. SHRs.

In addition to adjusting blood pressure, CaSR could regulate the function of VSMCs, such as proliferation, apoptosis, and calcification [4, 19]. However, little is known about the molecular mechanisms of CaSR in aortic remodeling in SHRs. By using the approach of immunohistochemistry staining, we observed that CaSR was increased in the media of the aortas from SHRs compared with those in WKY rats. Protein expressions of CaSR and NLRP3 inflammasome complex, comprising of NLRP3, ASC, and caspase 1, were increased in SHRs. And both the levels of procaspase 1 and active caspase 1 were increased in SHRs suggesting that the activity of caspase 1 was increased in SHRs. Calhex 231 could abolish the expression of CaSR, NLRP3, ASC, and caspase 1 in SHRs (Figures 1(a)–1(g)). These findings indicate that NLRP3 inflammasome was activated in SHRs which might be regulated by CaSR.

The NLRP3-caspase 1 inflammasome cascade plays a role in cleaving pro-IL-1*β* and pro-IL-18 into their mature forms IL-1*β* and IL-18. Protein expressions of pro-IL-1*β*, IL-1*β*, and IL-18 were increased in SHRs compared with WKY rats and all of them were inhibited when treated with Calhex 231 (Figures 1(h)–1(k)). Meanwhile, we detected infiltration of macrophage in vascular adventitia and found that CD68^+^, CD11b^+^, and CD206^+^ macrophages infiltrated in vascular adventitia from SHRs, which was prevented by Calhex 231 (Figure S1). These findings suggest that CaSR plays a role in the production and activation of IL-1*β* and inflammatory cell infiltration in the aortas from SHRs.

### 3.3. Suppression of CaSR Prevented Aortic Fibrosis in SHRs

**Figure 2:**
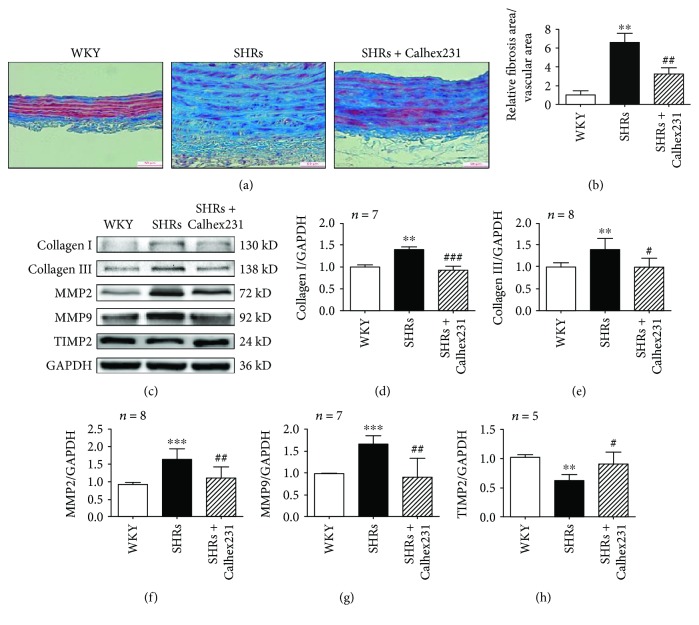
Suppression of CaSR prevented aortic fibrosis in SHRs. (a) Masson staining was used to detect aortic fibrosis, bar = 50 *μ*m, *n* = 5. (b) Quantification of the fibrosis area to vascular area of these arteries. (c) Western blots of collagen I, collagen III, MMP2, MMP9, and TIMP2 expressions. (d–h) Quantitative analyses of the proteins from C. ^∗∗^ *P* < 0.01 and ^∗∗∗^ *P* < 0.001 vs. WKY; ^#^ *P* < 0.05, ^##^ *P* < 0.01, and ^###^ *P* < 0.001 vs. SHRs.

To test whether CaSR is critical for aortic remodeling, we examined the effect of CaSR on aortic fibrosis. The ratio of fibrosis area to vascular area was increased in sections of aortas in SHRs compared with similar segments in WKY rats, and it was attenuated by Calhex 231 (Figures 2(a) and 2(b)). Meanwhile, collagen I and collagen III were increased in SHR aortas compared with those in WKY rats, and they were reduced when treated with Calhex 231. Modulation of collagen synthesis mainly depends on MMPs. MMP2 and MMP9 were upregulated in SHRs compared to WKY rats, and they were both downregulated by Calhex 231, which contrasts with tissue inhibitors of metalloproteinase 2 (TIMP2) (Figures 2(c)–2(h)). Together, these findings indicate that CaSR is associated with the synthesis and deposition of collagen and that it participates in aortic fibrosis in hypertension.

### 3.4. CaSR Activated the NLRP3 Inflammasome in VSMCs Induced by Ang II

**Figure 3:**
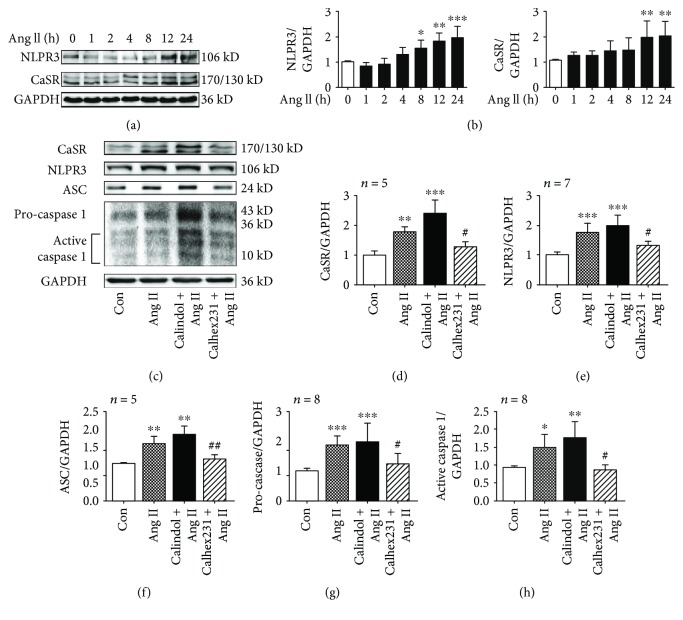
Calhex 231 prevented the activation of NLRP3 inflammasome in VSMCs induced by Ang II. (a) Western blot images of CaSR and NLRP3 expression induced by Ang II in a time-dependent manner. (b) Quantification of the proteins from A. ^∗^ *P* < 0.05, ^∗∗^ *P* < 0.01, and ^∗∗∗^ *P* < 0.001 vs. 0 h, *n* = 4. (c) CaSR regulated NLRP3 inflammasome activation induced by Ang II. Western blot images of CaSR, NLRP3, ASC, and caspase 1. (d–h) Quantitative analyses of the proteins from C. ^∗^ *P* < 0.05, ^∗∗^ *P* < 0.01, ^∗∗∗^ *P* < 0.001 vs. control; ^#^ *P* < 0.05 and ^##^ *P* < 0.01 vs. Ang II.

We found that the level of plasma Ang II from SHRs was increased (Figure S2). To identify potential pathways responsible for vascular remodeling, we cultivated VSMCs and incubated them with Ang II (10^−7^ mol/L) to mimic hypertension in vitro. The expression of NLRP3 and CaSR was increased after treatment with Ang II and changed with time, reaching a peak at 24 h (Figures 3(a) and 3(b)). It was speculated that Ang II increased NLRP3 inflammasome activity by activating CaSR in VSMCs. To verify this view, CaSR allosteric modulator was used before adding Ang II (10^−7^ mol/L, 24 h). Pretreatment of Calhex 231 (5 *μ*M for 30 min) and calindol (5 *μ*M for 1 h) was performed in this study. The levels of CaSR, NLRP3, ASC, procaspase 1, and active caspase 1 expression were increased after incubation with Ang II compared to the control group, while they were reduced by Calhex 231. Notably, the levels of above proteins were higher in VSMCs when treated with Ang II plus calindol, an agonist of CaSR, than those treated with Ang II only (Figures 3(c)–3(h)). These results demonstrated that CaSR triggered NLRP3 inflammasome activation in VSMCs induced by Ang II.

### 3.5. CaSR Contributes to IL-1*β* and IL-18 Release Induced by Ang II in VSMCs

**Figure 4:**
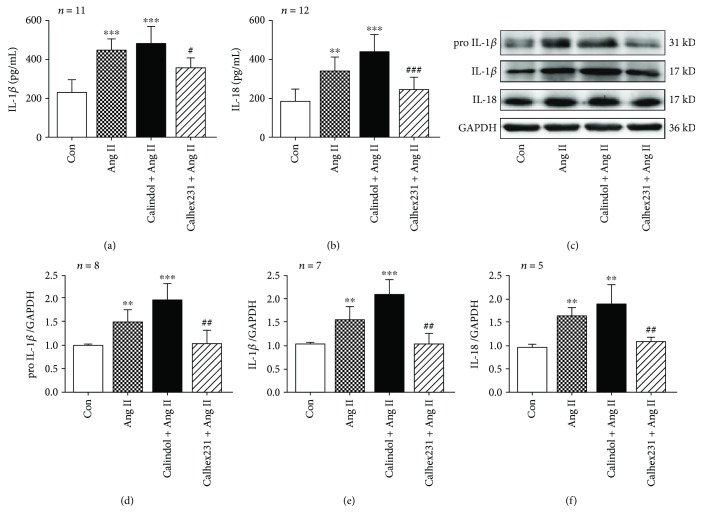
Calhex 231 inhibited the release of IL-1*β* and IL-18 in VSMCs induced by Ang II. (a, b) IL-1*β* and IL-18 levels measured with enzyme-linked immunosorbent assay (ELISA). (c) Western blot images of pro-IL-1*β*, IL-1*β*, and IL-18. (d–f) Quantitative analyses of the proteins from C. ^∗∗^ *P* < 0.01, ^∗∗∗^ *P* < 0.001 vs. control; ^#^ *P* < 0.05, ^##^ *P* < 0.01, and ^###^ *P* < 0.001 vs. Ang II.

Levels of proinflammatory cytokines IL-1*β* and IL-18 were increased in the supernatants of VSMCs induced by Ang II, which were inhibited by suppressing CaSR. Additionally, protein expressions of pro-IL-1*β*, IL-1*β*, and IL-18 were also enhanced in VSMCs incubated with Ang II and blocked by Calhex 231. Moreover, levels of pro-IL-1*β* and IL-1*β* were modestly higher when pretreated with calindol, an agonist of CaSR, compared with Ang II alone (Figure 4). Our findings indicate that Ang II could induce the activation of IL-1*β* and IL-18 in VSMCs and that could be modulated by CaSR.

### 3.6. CaSR Contributed to Collagen Synthesis in VSMCs Cultured with Ang II

**Figure 5:**
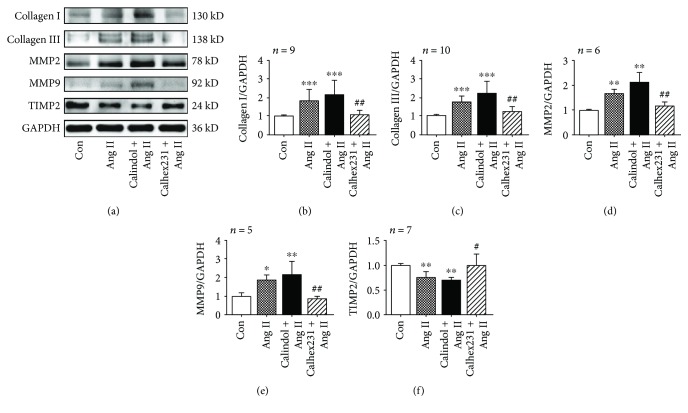
Calhex 231 suppressed collagen synthesis in VSMCs cultured with Ang II. (a) Western blots of collagen I, collagen III, MMP2, MMP9, and TIMP2 expressions. (b–f) Quantitative analysis of the proteins from A. ^∗^ *P* < 0.05, ^∗∗^ *P* < 0.01, and ^∗∗∗^ *P* < 0.001 vs. control; ^#^ *P* < 0.05 and ^##^ *P* < 0.01 vs. Ang II.

Collagen synthesis in VSMCs is a key point of aortic fibrosis. Then, we test the effects of pharmacological antagonism of CaSR on collagen synthesis. As shown in Figure 5, collagens I and III were induced by Ang II compared with the control group, while they were attenuated by Calhex 231. Moreover, the expression levels of MMP2 and MMP9 were increased after treatment with Ang II, and they were decreased by inhibiting CaSR. In addition, a lower level of TIMP2 in VSMCs incubated with Ang II was observed compared with the control group, which was reversed by Calhex 231, suggesting the importance of CaSR in collagen synthesis. Together, these findings suggest that CaSR contributed to collagen synthesis by activating MMP activity in VSMCs treated with Ang II and may be involved in aortic remodeling in hypertension.

### 3.7. CRID3 Inhibits NLRP3 Inflammasome Activity in VSMCs Induced by Ang II

**Figure 6:**
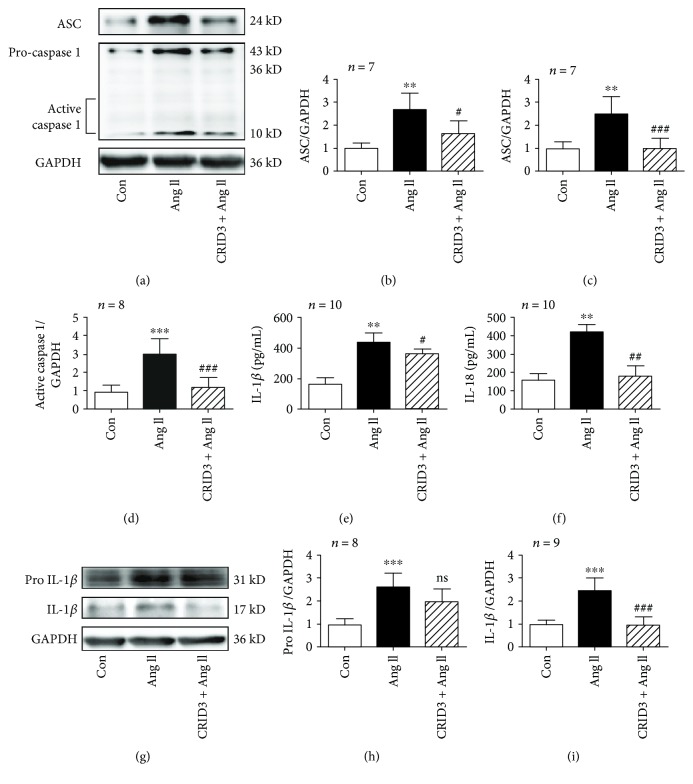
CRID3 inhibited the formation of NLRP3 inflammasomes and production of IL-1*β* and IL-18 in VSMCs stimulated with Ang II. (a) CRID3 (25 *μ*M) pretreatment for 30 min prevented increase in ASC and caspase 1 induced by Ang II. The analysis tables are shown in (b–d). (e, f) IL-1*β* and IL-18 in supernatants were analyzed by ELISA. Protein expressions of pro-IL-1*β* and IL-1*β* in VSMCs detected by western blot are shown in (g). (h, i) Bar graphs showed increased expression of pro-IL-1*β* and IL-1*β* induced by Ang II. CRID3 prevented the release of mature IL-1*β*, but failed to decrease pro-IL-1*β*. ^∗∗^ *P* < 0.01 and ^∗∗∗^ *P* < 0.001 vs. control; ^#^ *P* < 0.05, ^##^ *P* < 0.01, ^###^ *P* < 0.001, and ^ns^ *P* > 0.05 vs. Ang II.

CRID3 is a cytokine release inhibitor which is considered to be an inhibitor of NLRP3 inflammasome. CRID3 was used to test the effects of NLRP3 inflammasome on hypertensive vascular remodeling in vitro. ASC and caspase 1, including procaspase 1 and active caspase 1, induced by Ang II was suppressed by pretreatment of CRID3 (25 *μ*M for 30 min) as well as levels of IL-1*β* and IL-18 in supernatants. However, CRID3 failed to decrease the protein expression of pro-IL-1*β* induced by Ang II like IL-1*β* (Figure 6). These findings suggest that inhibiting NLRP3 inflammasome could suppress the release of cytokines IL-1*β* and IL-18 but not decrease the production of pro-IL-1*β*. NLRP3 inflammasome plays a role in the maturation of IL-1*β* and IL-18 in VSMCs induced by Ang II.

### 3.8. CRID3 Inhibits Collagen Synthesis in VSMCs Induced by Ang II

**Figure 7:**
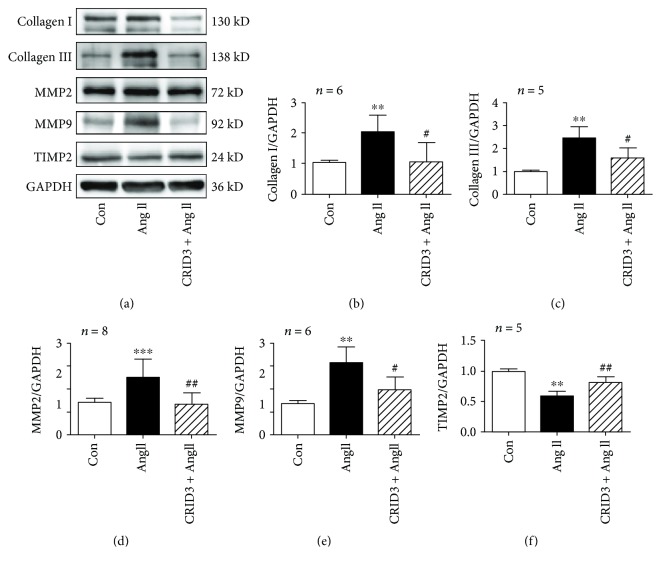
CRID3 inhibited collagen synthesis in VSMCs stimulated with Ang II. (a) Protein expression of collagen I, collagen III, MMP2, MMP9, and TIMP2 was detected by western blot. VSMCs were treated with CRID3 (25 *μ*M) for 30 min before adding Ang II. ^∗∗^ *P* < 0.01 and ^∗∗∗^ *P* < 0.001 vs. control; ^#^ *P* < 0.05 and ^##^ *P* < 0.01 vs. Ang II.

To examine whether CRID3 has an effect on collagen synthesis in VSMCs, protein expression of collagens I and III was detected. In VSMCs, CRID3 inhibited collagen synthesis induced by Ang II and CRID3 prevented the increased expression of MMP2 and MMP9 unlike TIMP2 (Figure 7), indicating that suppressing NLRP3 inflammasome was a target in aortic fibrosis in hypertension.

### 3.9. Calhex 231 Prevented Ang II-Induced Increase in Cytosolic [Ca^2+^]_i_ in VSMCs

Cytosolic [Ca^2+^]_i_ regulated by CaSR plays an important role in cellular signaling, which could stimulate NLRP3 inflammasome. To test whether NLRP3 inflammasome activation is [Ca^2+^]_i_ dependent or not, cytosolic [Ca^2+^]_i_ was detected. Ang II stimulated an increase [Ca^2+^]_i_ in VSMCs, which was attenuated by pretreatment of Calhex 231 and BAPTA/AM unlike CRID3. Stimulation of CaSR by calindol enhanced Ang II-induced [Ca^2+^]_i_ increase. These findings in part indicated that CaSR induced the increase of [Ca^2+^]_i_ flux that led to activation of NLRP3 inflammasome.

## 4. Discussion

In the present study, we demonstrated that CaSR and NLRP3 inflammasome were activated in the aortas from SHRs and VSMCs incubated with Ang II. And NLRP3 inflammasome was involved with CaSR-mediated fibrosis during hypertension, which led to IL-1*β* and IL-18 release.

**Figure 8:**
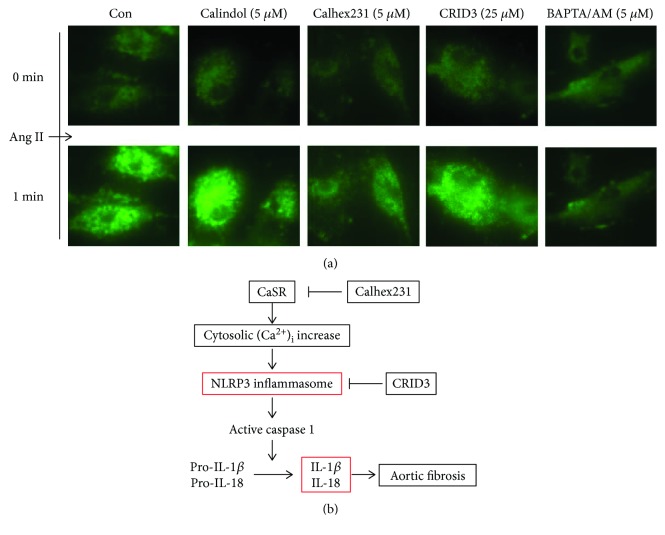
Calhex 231 pretreatment inhibits Ang II-induced increase in cytosolic [Ca^2+^]_i_ in VSMCs. (a) The cytosolic [Ca^2+^]_i_ was stained with fluo3/AM and the levels of cytosolic [Ca^2+^]_i_ were represented by intensity of fluorescence. Ang II induced an increase in cytosolic [Ca^2+^]_i,_ which was prevented when pretreatment of Calhex 231 and BAPTA/AM, *n* ≥ 10. (b) The schematic diagram of the mechanism of CaSR and NLRP3 inflammasome in vascular remodeling during hypertension. In VSMCs, cytosolic [Ca^2+^]_i_ increase induced by CaSR triggers NLRP3 inflammasome activation; NLRP3 inflammasome induces cytokines IL-1*β* and IL-18 release, which leads to aortic fibrosis.

Vascular inflammation and immune system promote the pathogenesis of vascular diseases such atherosclerosis and hypertension [20, 21]. Elevation of cytokines IL-1*β* was observed in experimental hypertension animals [6, 22]. However, the mechanism is not well understood. The study provides a new insight in hypertensive vascular remodeling, for which the proposed diagram is shown in Figure 8(b). In this model, it is speculated that CaSR activation in VSMCs increases cytosolic [Ca^2+^]_i_, which stimulates NLRP3 inflammasome activation and leads to IL-1*β* and IL-18 maturation during hypertensive aortic fibrosis. To test this hypothesis, we used SHRs and VSMCs stimulated by Ang II. Both of them were models of hypertensive vascular fibrosis. We designed the experiments by using CaSR allosteric modulator and NLRP3 inflammasome antagonist. The expression of CaSR in SHRs and VSMCs stimulated by Ang II was increased as well as NLRP3 inflammasome and fibrosis, indicating that CaSR may be a general joint of vascular inflammation during vascular fibrosis. To determine the role of CaSR in hypertension, we treated SHRs with Calhex 231 and found that the increased levels of blood pressure in SHRs were attenuated. Calhex 231 suppressed the activation of NLRP3 inflammasome in the aortas of SHRs and VSMCs induced by Ang II, indicating that CaSR regulates NLRP3 inflammasome in VSMCs.

NLRP3 inflammasome, comprising NLRP3, ASC, and caspase 1, promotes procaspase 1 (p40) into its active forms p10 and p20 [7]. In this study, expression of NLRP3, ASC, and caspase 1 was increased in the aortas from SHRs compared with those from WKY rats and Ang II-induced VSMCs, which was attenuated by Calhex 231. And we found that the level of plasma Ang II from SHRs was increased. Recent studies have shown that Ang II may contribute to vascular inflammation by activating the NLRP3 inflammasome via mitochondrial oxidative stress [22]. Ang II could induce hypertension during pregnancy in ASC^−/−^ mice but not in NLRP3^−/−^ mice, suggesting that NLRP3 contributes to hypertension independently of the inflammasomes [23]. Although studies have emphasized the importance of the NLRP3 inflammasome in hypertension, the exact mechanisms underlying NLRP3 inflammasome and its involvement in aortic remodeling in essential hypertension remain unknown. To test whether NLRP3 inflammasome is a critical part in VSMCs, CRID3 was used in this study. CRID3 is considered to be an inhibitor of NLRP3 inflammasome which inhibits caspase 1 activation and IL-1*β* processing by preventing ASC oligomerization [24]. In the present study, CRID3 inhibited ACS, caspase 1, and IL-1*β* processing stimulated by Ang II in VMSCs as well as collagen synthesis, indicating that NLRP3 inflammasome plays an important role in vascular fibrosis. Previous study has shown that knocking out NLRP3 inflammasome prevented VSMC phenotypic modulation in SHRs [25]. Comprehensively analyzing the above data, we propose that the activation of NLRP3 inflammasome in smooth muscle cells is coupled with vascular remodeling during hypertension.

Previous studies have reported that proinflammatory cytokines, IL-1*β* and IL-18, downstream targets of NLRP3 inflammasome, participate in the pathophysiology of cardiovascular disease and hypertension [26]. IL-1 family cytokines are considered upstream of other inflammatory cytokines, such as IL-6, IL-2, and IL-12 that are stimulated by IL-1*β* and IL-18, which are mainly secreted by inflammatory cells [26, 27]. However, other cell types, such as vascular endothelial cells and VSMCs, may also generate IL-1*β* under certain conditions [28, 29]. IL-1*β* was reported to evoke contractile activity and increase peripheral vascular resistance via phenylephrine [30]. Although the relationship between IL-18 and blood pressure is not clear, IL-18 could also promote the proliferation and migration of VSMCs [31]. IL-1*β* and IL-18 were also reported to contribute cardiac remodeling by promoting fibrosis [16]. Consistent with previous study, the inhibition of NLRP3 inflammasome by CRID3 decreased the levels of IL-1*β* and IL-18 same as the expression levels of collagen I, collagen III, MMP2, and MMP9. These findings indicate that NLRP3 inflammasome may promote the remodeling of extracellular matrix via stimulating IL-1*β* and IL-18.

We also observed that the levels of IL-1*β*, IL-18, collagens, and MMPs in the aortas from SHRs and VSMCs induced by Ang II were also attenuated by inhibiting CaSR. Release of IL-1*β* and IL-18 in VSMCs could contribute to the accumulation of inflammatory cells like macrophages [26, 32]. By using the approach of immunohistochemistry, we detected infiltration of macrophages in vascular adventitia and found that macrophages infiltrated in vascular adventitia from SHRs, which was prevented by Calhex 231. In line with previous studies, VSMCs secrete IL-1*β*, and this, in turn, leads to accumulate macrophages and contribute to vascular lesions [25, 29]. These results suggest that inhibiting CaSR prevents macrophage infiltration, proinflammatory cytokine release, and collagen synthesis. Moreover, the production of pro-IL-1*β* in the aortas from SHRs and VMSCs incubated with Ang II was reduced by Calhex 231 unlike those treated with CRID3, indicating that other mechanisms besides NLRP3 inflammasome are involved in CaSR-mediated aortic remodeling, which need to be further studied.

It was speculated that CaSR was correlated with Ang II in SHRs [15]; however, no research has ever been conducted to verify the direct effect of the two. To investigate the relationship between CaSR and Ang II, we used Ang II to stimulate VSMCs directly in vitro. Then, we found that CaSR was increased in VSMCs treated with Ang II and changed with time, while it was blocked by Calhex 231. CaSR, a modulator of blood pressure and vascular tone, is functionally expressed in VSMCs, endothelial cells, parathyroid cells, and perivascular nerves [10]. Several studies have reported that calcimimetics, agonists of CaSR, decrease blood pressure in uremic rats and SHRs but not in normotensive rats [33, 34]. Conversely, calcilytic NPS 2143, an antagonist of CaSR, increases blood pressure in normotensive rats in the presence of parathyroid glands [35]. However, a later study demonstrated that NPS 2143 failed to increase the blood pressure in the presence of calcium channel inhibitor and type 1 Ang II receptor antagonist [36]. Our previous study has reported that the level of blood pressure in SHRs was decreased by inhibiting CaSR and mitochondrial fission [37]. In line with our findings, study was performed recently and found that elevation of the blood pressure and endothelium-independent relaxation were observed with the loss of CaSR-mediated contraction in VMSCs in the aorta and mesenteric artery in response to KCl and phenylephrine in ^SM22A^CaSR^△flox/△flox^ mice [14]. In contrast, CaSR induced endothelium-dependent vasorelaxations mediated by nitric oxide and K^+^ channels in the mesenteric arteries [38], suggesting that the regulation of vascular contractility is a local balance between VSMCs and endothelium other than hormonal alteration. What worth noting is that Calhex 231 reduced the activation of NLRP3 inflammasome and IL-1*β*, which probably lead to the downregulation of blood pressure in SHRs. Calhex 231, a negative allosteric modulator of CaSR, blocks inositol phosphates and decreases Ca^2+^ concentrations [39]. Calindol, a positive allosteric modulator of CaSR, revealed as synergistic for Ca^2+^ site on the 7TMD [40]. There is a positive feedback regulation between CaSR and Ca^2+^ that is CaSR increases Ca^2+^, which in turn activates CaSR and its expression [16]. Fluo-3/AM is a fluorescent probe that indicates cytosolic [Ca^2+^]_i_. An increase [Ca^2+^]_i_ was observed in VSMCs stimulated with Ang II, which was attenuated by pretreatment of Calhex 231 and BAPTA/AM unlike CRID3. And calindol pretreatment enhanced Ang II-induced [Ca^2+^]_i_ increase. These findings at least partially indicated that the increased [Ca^2+^]_i_ flux induced by CaSR led to the activation of NLRP3 inflammasome.

## 5. Conclusions

In summary, our findings demonstrated that NLRP3 inflammasome and CaSR were induced by Ang II in VSMCs and increased in the aortas of SHRs. Additionally, CaSR participated in aortic remodeling via regulating blood pressure levels, activating NLRP3-caspase 1 inflammasome and fibrosis. These findings have important clinical implications and suggest that inhibition of CaSR-NLRP3 inflammasome may be a therapeutic target for cardiovascular disease.

## Figures and Tables

**Table 1:** Effect of Calhex 231 on blood pressure, heart rate, and body weight in SHRs.

	WKY	SHRs	SHRs+Calhex 231
SBP (mmHg)	100.56 ± 8.48	227.83±2.55^∗∗∗^	201.21 ± 8.50^Δ#^
DBP (mmHg)	84.12 ± 12.70	203.94±2.75^∗∗∗^	179.35 ± 8.50^Δ#^
MAP (mmHg)	89.66 ± 11.23	211.96±2.76^∗∗∗^	186.74 ± 8.46^Δ#^
HR (bpm)	288.76 ± 36.29	431.62±30.93^∗∗∗^	426.28 ± 27.07^Δ★^
Body weight (g)	315.72 ± 30.28	317.33 ± 15.88^▲^	325.53 ± 19.78^★^

Note: ^∗∗∗^ *P* < 0.001 vs. WKY, ^Δ^ *P* < 0.001 vs. WKY, ^#^ *P* < 0.01 vs. SHRs, ^▲^ *P* > 0.05 vs. WKY. ^★^ *P* > 0.05 vs. SHRs, *n* ≥ 4.

## Data Availability

All relevant data are within the paper and its Supporting Information files.

## Supplementary Materials

Supplementary Materials Figure S1: infiltration of macrophage in vascular adventitia of the aortas from SHRs. Immunohistochemistry was used to detect CD68, CD11b, and CD206. The positive cells were shown as brown-stained, *n* = 5. Figure S2: plasma levels of Ang II increased in SHRs. ELISA was used to detect the levels of Ang II in plasma, ^∗∗∗^ *P* < 0.01 vs. WKY rats, *n* ≥ 7.

## References

[1] Intengan H. D., Schiffrin E. L. (2001). Vascular remodeling in hypertension: roles of apoptosis, inflammation, and fibrosis. *Hypertension*.

[2] Renna N. F., de Las Heras N., Miatello R. M. (2013). Pathophysiology of vascular remodeling in hypertension. *International Journal of Hypertension*.

[3] Wu D., Ren P., Zheng Y. (2017). NLRP3 (nucleotide oligomerization domain-like receptor family, pyrin domain containing 3)-caspase-1 inflammasome degrades contractile proteins: implications for aortic biomechanical dysfunction and aneurysm and dissection formation. *Arteriosclerosis, Thrombosis, and Vascular Biology*.

[4] Wen C., Yang X., Yan Z. (2013). Nalp3 inflammasome is activated and required for vascular smooth muscle cell calcification. *International Journal of Cardiology*.

[5] Usui F., Shirasuna K., Kimura H. (2012). Critical role of caspase-1 in vascular inflammation and development of atherosclerosis in Western diet-fed apolipoprotein E-deficient mice. *Biochemical and Biophysical Research Communications*.

[6] Qi J., Yu X. J., Shi X. L. (2016). NF-*κ*B blockade in hypothalamic paraventricular nucleus inhibits high-salt-induced hypertension through NLRP3 and caspase-1. *Cardiovascular Toxicology*.

[7] Latz E., Xiao T. S., Stutz A. (2013). Activation and regulation of the inflammasomes. *Nature Reviews Immunology*.

[8] Zhang B., Liu Y., Sui Y. B. (2015). Cortistatin inhibits NLRP3 inflammasome activation of cardiac fibroblasts during sepsis. *Journal of Cardiac Failure*.

[9] Tfelt-Hansen J., Schwarz P., Brown E. M., Chattopadhyay N. (2003). The calcium-sensing receptor in human disease. *Frontiers in Bioscience*.

[10] Smajilovic S., Yano S., Jabbari R., Tfelt-Hansen J. (2011). The calcium-sensing receptor and calcimimetics in blood pressure modulation. *British Journal of Pharmacology*.

[11] Alfadda T. I., Saleh A. M. A., Houillier P., Geibel J. P. (2014). Calcium-sensing receptor 20 years later. *American Journal of Physiology-Cell Physiology*.

[12] Zhong X., Wang Y., Wu J. (2015). Calcium sensing receptor regulating smooth muscle cells proliferation through initiating cystathionine-gamma-lyase/hydrogen sulfide pathway in diabetic rat. *Cellular Physiology and Biochemistry*.

[13] Rossol M., Pierer M., Raulien N. (2012). Extracellular Ca^2+^ is a danger signal activating the NLRP3 inflammasome through G protein-coupled calcium sensing receptors. *Nature Communications*.

[14] Schepelmann M., Yarova P. L., Lopez-Fernandez I. (2016). The vascular Ca^2+^-sensing receptor regulates blood vessel tone and blood pressure. *American Journal of Physiology-Cell Physiology*.

[15] Qu Y. Y., Hui J., Wang L. M. (2016). Reduced expression of the extracellular calcium-sensing receptor (CaSR) is associated with activation of the renin-angiotensin system (RAS) to promote vascular remodeling in the pathogenesis of essential hypertension. *PLoS One*.

[16] Liu W., Zhang X., Zhao M. (2015). Activation in M1 but not M2 macrophages contributes to cardiac remodeling after myocardial infarction in rats: a critical role of the calcium sensing receptor/NRLP3 inflammasome. *Cellular Physiology and Biochemistry*.

[17] Montezano A. C., Lopes R. A. M., Neves K. B., Rios F., Touyz R. M. (2017). Isolation and culture of vascular smooth muscle cells from small and large vessels. *Methods in Molecular Biology*.

[18] Molostvov G., Fletcher S., Bland R., Zehnder D. (2008). Extracellular calcium-sensing receptor mediated signalling is involved in human vascular smooth muscle cell proliferation and apoptosis. *Cellular Physiology and Biochemistry*.

[19] Henaut L., Boudot C., Massy Z. A. (2014). Calcimimetics increase CaSR expression and reduce mineralization in vascular smooth muscle cells: mechanisms of action. *Cardiovascular Research*.

[20] Ryan M. J. (2013). An update on immune system activation in the pathogenesis of hypertension. *Hypertension*.

[21] Schiffrin E. L. (2014). Immune mechanisms in hypertension and vascular injury. *Clinical Science*.

[22] Usui F., Shirasuna K., Kimura H. (2015). Inflammasome activation by mitochondrial oxidative stress in macrophages leads to the development of angiotensin II-induced aortic aneurysm. *Arteriosclerosis, Thrombosis, and Vascular Biology*.

[23] Shirasuna K., Karasawa T., Usui F. (2015). NLRP3 deficiency improves angiotensin II-induced hypertension but not fetal growth restriction during pregnancy. *Endocrinology*.

[24] Coll R. C., Robertson A., Butler M., Cooper M., O'Neill L. A. (2011). The cytokine release inhibitory drug CRID3 targets ASC oligomerisation in the NLRP3 and AIM2 inflammasomes. *PLoS One*.

[25] Sun H. J., Ren X. S., Xiong X. Q. (2017). NLRP3 inflammasome activation contributes to VSMC phenotypic transformation and proliferation in hypertension. *Cell Death and Disease*.

[26] Krishnan S. M., Sobey C. G., Latz E., Mansell A., Drummond G. R. (2014). IL-1*β* and IL-18: inflammatory markers or mediators of hypertension?. *British Journal of Pharmacology*.

[27] Mills K. H. G., Dungan L. S., Jones S. A., Harris J. (2013). The role of inflammasome-derived IL-1 in driving IL-17 responses. *Journal of Leukocyte Biology*.

[28] Dewberry R., Holden H., Crossman D., Francis S. (2000). Interleukin-1 receptor antagonist expression in human endothelial cells and atherosclerosis. *Arteriosclerosis, Thrombosis, and Vascular Biology*.

[29] Tangi T. N., Elmabsout A. A., Bengtsson T., Sirsjo A., Fransen K. (2012). Role of NLRP3 and CARD8 in the regulation of TNF-*α* induced IL-1*β* release in vascular smooth muscle cells. *International Journal of Molecular Medicine*.

[30] Dorrance A. M. (2007). Interleukin 1-beta (IL-1*β*) enhances contractile responses in endothelium-denuded aorta from hypertensive, but not normotensive, rats. *Vascular Pharmacology*.

[31] Valente A. J., Yoshida T., Murthy S. N. (2012). Angiotensin II enhances AT1-Nox1 binding and stimulates arterial smooth muscle cell migration and proliferation through AT1, Nox1, and interleukin-18. *American Journal of Physiology-Heart and Circulatory Physiology*.

[32] Harwani S. C. (2018). Macrophages under pressure: the role of macrophage polarization in hypertension. *Translational Research*.

[33] Odenwald T., Nakagawa K., Hadtstein C. (2006). Acute blood pressure effects and chronic hypotensive action of calcimimetics in uremic rats. *Journal of the American Society of Nephrology*.

[34] Ogata H., Ritz E., Odoni G., Amann K., Orth S. R. (2003). Beneficial effects of calcimimetics on progression of renal failure and cardiovascular risk factors. *Journal of the American Society of Nephrology*.

[35] Rybczynska A., Lehmann A., Jurska-Jasko A. (2006). Hypertensive effect of calcilytic NPS 2143 administration in rats. *The Journal of Endocrinology*.

[36] Rybczynska A., Jurska-Jasko A., Boblewski K., Lehmann A., Orlewska C. (2010). Blockade of calcium channels and AT1 receptor prevents the hypertensive effect of calcilytic NPS 2143 in rats. *Journal of Physiology and Pharmacology*.

[37] Zhang X., Chen W., Li J. (2018). Involvement of mitochondrial fission in calcium sensing receptor-mediated vascular smooth muscle cells proliferation during hypertension. *Biochemical and Biophysical Research Communications*.

[38] Greenberg H. Z. E., Shi J., Jahan K. S. (2016). Stimulation of calcium-sensing receptors induces endothelium-dependent vasorelaxations via nitric oxide production and activation of IKCa channels. *Vascular Pharmacology*.

[39] Petrel C., Kessler A., Maslah F. (2003). Modeling and mutagenesis of the binding site of Calhex 231, a novel negative allosteric modulator of the extracellular Ca^2+^-sensing receptor. *Journal of Biological Chemistry*.

[40] Ray K., Tisdale J., Dodd R. H., Dauban P., Ruat M., Northup J. K. (2005). Calindol, a positive allosteric modulator of the human Ca^2+^ receptor, activates an extracellular ligand-binding domain-deleted rhodopsin-like seven-transmembrane structure in the absence of Ca^2+^. *Journal of Biological Chemistry*.

