# Supplementary material for: NLRP3 Inflammasome Is Involved in Calcium-Sensing Receptor-Induced Aortic Remodeling in SHRs
Source: Mediators Inflamm. 2019 Feb 13;2019:6847087. doi: 10.1155/2019/6847087 (PMC6393924; doi:10.1155/2019/6847087)
Supplement: Supplementary Materials — Figure S1: infiltration of macrophage in vascular adventitia of the aortas from SHRs. Immunohistochemistry was used to detect CD68, CD11b, and CD206. The positive cells were shown as brown-stained, n = 5. Figure S2: plasma levels of Ang II increased in SHRs. ELISA was used to detect the levels of Ang II in plasma, ∗∗∗ P < 0.01 vs. WKY rats, n ≥ 7. [file 6847087.f1.pdf]

SUPPLEMENTAL

Inhibition of NLRP3 inflammasome by suppressing calcium sensing receptor prevents aortic remodeling in hypertension

Xin Zhang, Siting Hong, Shuhan Qi, Wenxiu Liu, Xiaohui Zhang, Zhiyu Shi, Wenjia Chen, Meng Zhao, Xinhua Yin*

Department of Cardiology, First Affiliated Hospital of Harbin Medical University, Harbin 150001, Heilongjiang, P. R. China

***Corresponding author:**

Xinhua Yin, MD, PhD

Department of Cardiology

First Affiliated Hospital of Harbin Medical University

No.23, YouZheng Street, NanGang District, Harbin150001

Heilongjiang Province, China

E-mail: yinxinhua5063@163.com

Materials and Methods

Infiltration of macrophage in the aortas from experimental rats

Immunohistochemistry staining was used to detected infiltration of macrophage in the vascular adventitia. Antibodies against CD68, CD11b, and CD206 represented

mononuclear macrophages, M1 macrophage subtypes, and M2 macrophage subtypes, respectively. Antibodies used in this study were as follows: CD68 (1:50, Abacm), CD11b (1:400, Abcam), and CD206 (1:100, Santa Cruz). These images were captured by using a microscope (Zeiss, Germany). Cells with brown-strained particles were considered to be positive.

Measurement of angiotensin II concentration in plasma from experimental tats

Levels of Ang II in plasma were measured using an Elisa kit (Nanjing Jiancheng Bioengineering Institute, Nanjing, China) according to the manufacturer's instructions. Briefly, adult male WKY rats and SHRs were sacrificed after anesthetized by 10% chloralic hydras. Blood samples were collected and centrifuged at 3,000rpm for 15 min at 4℃ and the plasma were obtained. Plasma levels of Ang II were determined by standard commercially available enzyme immunoassay. Optical density was read at 450 nm using a Microplate Reader (BioTek, USA).

Results

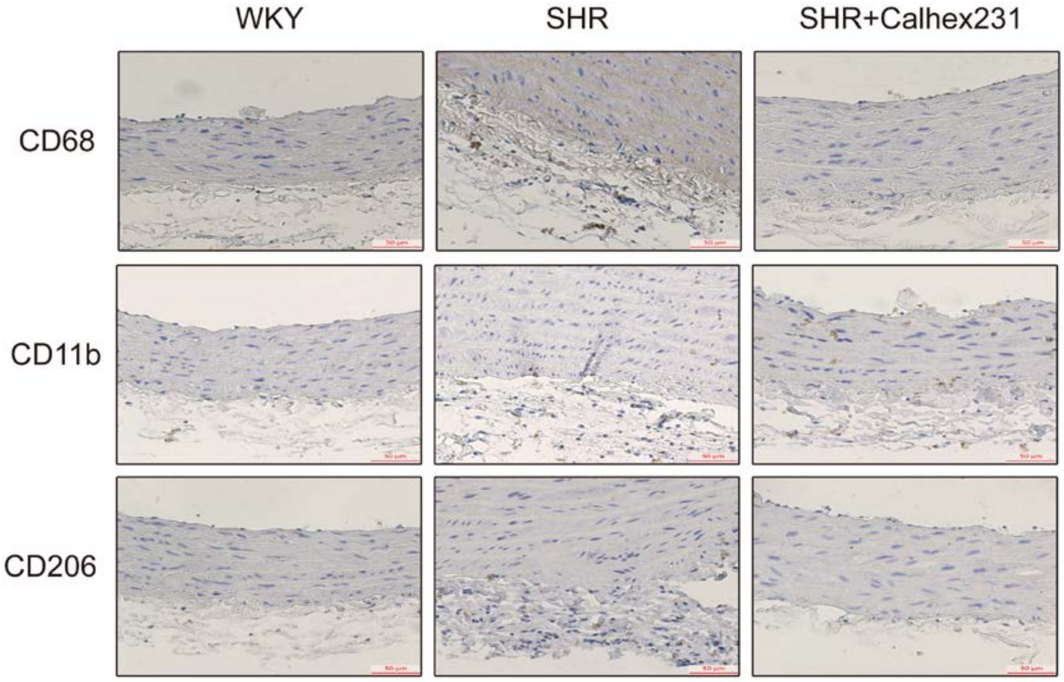

Figure S1. Infiltration of macrophage in vascular adventitia of the aortas from SHRs. Immunohistochemistry was used to detect CD68, CD11b, and CD206. The positive cells were shown as brown-stained, n = 5.

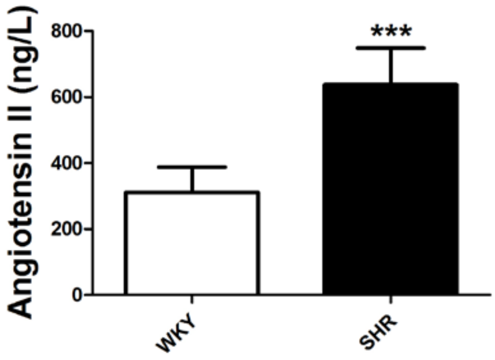

Figure S2. Plasma levels of Ang II increased in SHRs. Elisa was used to detect the levels of Ang II in plasma, *** $P < 0.01$ *vs.* WKY rats, n ≥ 7.
